# Extracts of Dunkelfelder Grape Seeds and Peel Increase the Metabolic Rate and Reduce Fat Deposition in Mice Maintained on a High-Fat Diet

**DOI:** 10.3390/foods12173251

**Published:** 2023-08-29

**Authors:** Chenlu Yang, Xuelin Tian, Yulei Han, Xueqing Shi, Hua Wang, Hua Li

**Affiliations:** 1 College of Enology, Northwest A&F University, Yangling 712100, China; yclwine@nwsuaf.edu.cn (C.Y.); tianxuelin@nwafu.edu.cn (X.T.); hanyl@nwafu.edu.cn (Y.H.); shixueqingkkaa@163.com (X.S.); wanghua@nwsuaf.edu.cn (H.W.); 2 China Wine Industry Technology Institute, Yinchuan 750021, China; 3 Shaanxi Engineering Research Center for Viti-Viniculture, Yangling 712100, China

**Keywords:** energy expenditure, fat accumulation, grape pomace, high-fat diet, lipid metabolism, polyphenol, thermogenesis

## Abstract

Chronic high-fat diet intake may induce obesity and increase the risk of metabolic syndrome. The pomace of grape (*Vitis vinifera* L.) is rich in polyphenols, which are candidates for anti-obesity therapy. The present study aimed to investigate the effects of Dunkelfelder grape seed extract (GSE) and grape peel extract (GPE) on lipid and energy metabolism disorders in mice maintained on a high-fat diet (HFD). Male nine-week C57BL/6J mice were randomly assigned to one of four groups, namely, the normal chow diet (ND), HFD, HFD plus GSE (400 mg/kg BW) administered by oral gavage, or HFD plus GPE (400 mg/kg BW) administered by oral gavage. There were eight mice per group, and the experiment was 14 weeks in duration. The results showed that GSE and GPE treatments did not affect energy intake in mice on a high-fat diet, but body weight gain was 24.5% and 17.3% lower in the GSE- and GPE-treated mice than in the HFD group, respectively. They also decreased blood triglyceride (TG), total cholesterol (TC), and fasting blood glucose levels and increased high-density lipoprotein cholesterol (HDL-C). In addition, GSE and GPE reduced adipose tissue weight and excessive lipid droplet accumulation in the adipocytes. The metabolic chamber test showed that the GSE and GPE treatments enhanced oxygen consumption, carbon dioxide production, and heat release while decreasing the respiratory exchange rate (RER). This suggests that GSE and GPE augmented fuel oxidation and energy generation and increased the proportion of lipids being utilized in energy metabolism. GSE and GPE also upregulated the genes controlling lipolysis and downregulated those controlling lipogenesis in adipose tissues. Moreover, they significantly increased the expression levels of the genes regulating thermogenesis in BAT, eWAT, and iWAT, and mitochondrial biogenesis in all three types of adipose tissue. In conclusion, the present study empirically demonstrated that GSE and GPE enhance body fat utilization by augmenting lipid and energy metabolism and could, therefore, ameliorate high-fat diet-induced obesity.

## 1. Introduction

In recent decades, the human dietary habit has begun to shift towards a high-energy diet rich in fats and sugars, especially in developing countries [1]. When energy consumption exceeds energy expenditure for an extended period, metabolism is dysregulated, and obesity occurs [2]. Excessive fat accumulation is a major characteristic of obesity, and the latter has become an important global public health problem [3]. Obesity increases the risk of hypertension, dyslipidemia, cardiovascular disease, and type 2 diabetes mellitus [4,5,6,7].

Increasing energy expenditure and augmenting the basal metabolic rate have become important treatment options for obesity and overweight. Recent studies on rodents revealed that inducing thermogenesis in brown adipose tissue (BAT) or converting white adipocytes to beige adipocytes in white adipose tissue (WAT) could also potentially balance metabolic energy [8,9] as these mechanisms increase the number of mitochondria in BAT and WAT. Moreover, mitochondrial uncoupling protein 1 (UCP1) in the inner membranes of the mitochondria in brown and beige adipocytes uncouples mitochondrial respiration from ATP biosynthesis and dissipates thermal energy (heat) [10,11]. This process is associated with enhanced fatty acid and glucose consumption and, by extension, an increase in energy substrate metabolism in adipose tissue [10].

Grape, the berry of *Vitis vinifera* L., has economic importance due to its uses in the production of wine, grape juice, and other foods. Winemaking generates a residue known as grape pomace. This material accounts for ~20–25% of the total grape mass and consists of peel, seeds, and traces of pulp [12]. Regular consumption of a diet containing an appropriate amount of grape pomace may help in the reduction of body fat accumulation and the prevention of obesity [13]. This benefit may be related to the fact that grape pomace is rich in phenolic substances (such as proanthocyanidins, anthocyanins, resveratrols, etc.), which have various physiological functions and may mitigate the health risks associated with metabolic syndrome [14,15,16]. Previous investigations on the anti-obesity effects of grape pomace or grape seed extract (GSE) have focused mainly on plasma profiles, glucose metabolism (insulin sensitivity), inflammation, hepatic steatosis, and adipocyte expansion [17,18,19,20]. However, few studies have attempted to elucidate whether the mechanisms by which GSE ameliorates the dysregulated lipid metabolism are related to the modulation of energy metabolism, and whether the thermogenesis and mitochondrial biogenesis in adipose tissue are regulated by GSE. Grape peel also contains comparatively low but important polyphenol levels. Nevertheless, the potential effects of grape peel extract (GPE) on energy regulation, WAT browning, and BAT thermogenesis are unknown.

Here, Dunkelfelder grape pomace GSE and GPE were selected as the plant material. We hypothesized that GSE and GPE supplementation could normalize lipid and energy metabolism in mice with HFD-induced obesity by augmenting the thermogenic capacity and mitochondrial biogenesis of adipose tissue. In the present work, we investigated the effects of GSE and GPE on body weight gain, energy intake, and serum lipid profiles in HFD-fed mice. We also examined the effects of GSE and GPE on the weight and the sizes of the adipocytes in BAT, epididymal white adipose tissue (eWAT), and inguinal white adipose tissue (iWAT). We evaluated the influences of GSE and GPE on the O_2_ consumption, CO_2_ production, heat generation, and respiratory exchange rate (RER) of mice in metabolic chambers. Finally, we investigated the impact of GSE and GPE on the expression levels of the genes regulating lipolysis and lipogenesis in BAT, eWAT, and iWAT, thermogenesis in BAT, browning in eWAT and iWAT, and mitochondrial biogenesis in all three types of adipose tissues.

## 2. Materials and Methods

### 2.1. Plant Material and Sample Preparation

The solid residue of *Vitis vinifera* L. (Dunkelfelder) used in the present study was obtained from the pressing step in winemaking and was provided by Caoxingzhuang vineyard (Yangling, China). Grape seeds and peel were manually sorted from fresh pomace, vacuum freeze-dried, and pulverized. Eighty grams of grape seed or peel powder was placed in the extraction tank of an accelerated solvent extractor (Dionex, Sunnyvale, CA, USA). The extraction tank contained diatomaceous earth which was mixed with each plant powder at a 1:1 (*v*/*v*) ratio. Each mixture was extracted with 70% (*v*/*v*) ethanol according to the methods of Yang et al. [21] and Monrad et al. [22], and solutions of the polyphenolic complexes in the grape seed and peel powders were produced. The solutions were centrifuged at 10,000 rpm and 4 °C for 10 min and the supernatants were collected, concentrated, and lyophilized. The freeze-dried grape seed and peel extracts were designated GSE and GPE, respectively, and were stored at −20 °C until the subsequent analyses.

### 2.2. Phenolic Compound Analyses

#### 2.2.1. Spectrophotometric Characterization

An ultraviolet–visible (UV–Vis) spectrophotometer (Infinite M200 Pro, Tecan, Mannedorf, Switzerland) was used to quantify the total phenols (TP), total tannin (TAN), total flavonoids (TFO), total flavanols (TFA) and total anthocyanins (TA) in the GSE and GPE. For this purpose, 3 mg of GSE or GPE was solubilized in 5 mL of a mixture of 60% (*v*/*v*) methanol and 0.1% (*v*/*v*) HCl.

The Folin–Ciocalteu method was used to determine the TP content [23]. In brief, the GSE or GPE solution was combined with Folin–Ciocalteu reagent, and sodium carbonate solution (10% *w*/*v*) was added to the mixture. The reaction proceeded in the dark at room temperature (20–25 °C) for 120 min, and the absorbance was measured in the UV–Vis spectrophotometer at 765 nm. The results were expressed in milligrams of gallic acid equivalents (GAE) per gram GSE or GPE. (A calibration curve was generated using this method and different concentrations of gallic acid solution: y = 0.1056x + 0.0528, r^2^ = 0.9996.)

The methylcellulose precipitation method was used to determine the TAN content [24]. GSE or GPE solution (0.1 mL) was added to two centrifuge tubes. Three milliliters of 0.04% (*w*/*v*) methylcellulose solution were added to the first, and none was added to the second. The contents of the first were mixed and allowed to react for 2–3 min. Then, 2 mL saturated ammonium sulfate solution was added to both tubes and diluted to 10 mL final volume with distilled water. The mixtures were left to react in the dark at room temperature for 120 min and centrifuged at 1000 rpm for 20 min. The absorbance of the supernatant was then measured at 280 nm in the UV–Vis spectrophotometer and the final tannin absorbance was calculated as the difference between the absorbances of the supernatants in the two centrifuge tubes. The results were expressed in milligrams of (+)-catechin equivalents (CE) per gram GSE or GPE. (A calibration curve was generated using this method and different concentrations of (+)-catechin solution: y = 0.0194x + 0.0034, r^2^ = 0.9998.)

The TFO content was determined according to the method of Peinado et al. [25]. In a centrifuge tube, 700 μL methanol, 2.7 mL of a 30% (*v*/*v*) methanol solution, 200 μL of 0.5 M NaNO_2_, and 200 μL of 0.3 M AlCl_3_ were added in sequence to 300 μL GSE or GPE solution. After 5 min, 1.0 mL of 1 M NaOH was added. After 10 min, the absorbance was measured in the UV–Vis spectrophotometer at 510 nm. The results were expressed in milligrams of rutin equivalents (RE) per gram GSE or GPE. (A calibration curve was generated using this method and different concentrations of rutin solution: y = 1.7453x + 0.0264, r^2^ = 0.9994.)

The TFA content was detected by the *p*-DMACA method [26]. GSE or GPE solution (0.1 mL) and 3 mL *p*-DMACA solution (0.1% (*v*/*v*) in 1 M HCl in methanol) were combined in a centrifuge tube. The reaction proceeded at room temperature for 10 min, and the absorbance was measured at 640 nm in the UV–Vis spectrophotometer. The results were expressed in milligrams of (+)-catechin equivalents per gram GSE or GPE. (A calibration curve was generated using this method and different concentrations of (+)-catechin solution: y = 0.0079x + 0.0043, r^2^ = 0.9997.)

The pH differential method was used to determine the TA content [27]. GPE solution was equally diluted with pH 1.0 or pH 4.5 buffer. The absorbances of both solutions were measured at 510 nm and 700 nm in the UV–Vis spectrophotometer. The results were expressed in milligrams of cyanidin-3-glucoside equivalents (C3GE) per gram GPE and calculated as follows:

*A* = (*A*_510_ − *A*_700_)_pH 1.0_ − (*A*_510_ − *A*_700_)_pH 4.5_ (1)

TA content = (*A* × MW × DF × *V_e_* × 1000) / (ε × 1 × *M*) (2)

where MW is the molecular weight of cyanidin-3-glucoside (449 g/mol), DF is the dilution factor, *V_e_* is the extract volume, ε is the molar extinction coefficient of cyanidin-3-glucoside (29,600), and *M* is the mass of the extracted peels.

#### 2.2.2. High-Performance Liquid Chromatography (HPLC) Analyses of the GSE Phenolic Profiles

An 80 mg amount of GSE was combined by vigorous manual mixing with 10 mL of 90% (*v*/*v*) aqueous ethyl acetate and left to stand for 30 min. The upper organic phase was collected and the foregoing extraction procedure was repeated. The organic phase was evaporated to dryness at 40 °C in a RapidVap evaporator (Labconco Corp., Kansas City, MO, USA). The residue was dissolved in 1 mL HPLC-grade methanol and passed through a 0.45 μm syringe filter. Target phenolics were quantitated in a high-performance liquid chromatography (HPLC) system (Shimadzu Corp., Kyoto, Japan) fitted with a C18 column (250 mm × 4.6 mm; Shimadzu Corp.) and a variable wavelength detector (VWD). The test parameters used were previously reported [28]. The mobile phase consisted of solutions A (1% (*v*/*v*) aqueous acetic acid) and B (1% (*v*/*v*) aqueous acetic acid-acetonitrile), the detection wavelength was 280 nm, the column temperature was 30 °C, the flow rate was 1 mL/min, and the gradient was 5–25% solution B for 40 min, 25–35% solution B for 5 min, and 35–50% solution B for 5 min.

#### 2.2.3. HPLC Analyses of the GPE Anthocyanin Profiles

Twenty-five milligrams of GPE were mixed with 5 mL of 2% (*v*/*v*) methanol, placed in an ultrasonic bath for 10 min, shaken for 30 min, and centrifuged at 8000 rpm for 5 min. The supernatants were collected, and the precipitate was extracted twice by the foregoing procedure. Then, 1 mL supernatant was evaporated to dryness at 40 °C in the RapidVap evaporator, and the residue was redissolved in 1 mL of solution A (32:4:1 (*v*/*v*/*v*) water/acetonitrile/methanoic acid). The extracts were then passed through a 0.45 μm syringe filter. The target anthocyanins were determined using HPLC coupled with a photodiode array detector (Shimadzu Corp., Suzhou, China) and a Synergi Hydro-RP C18 column (250 mm × 4.6 mm, 4 µm; Phenomenex, Torrance, CA, USA). The test parameters used were previously published [29]. The mobile phase consisted of solutions A and B (16:20:1 (*v*/*v*/*v*) water/acetonitrile/methanoic acid), the detection wavelength was 520 nm, the column temperature was 35 °C, the flow rate was 1 mL/min, and the gradient was 0–10% B at 0–15 min, 10–20% B at 15–30 min, 20–35% B at 30–45 min, 35–100% B at 46–50 min, and 100% B at 50–51 min.

### 2.3. Animals and Diets

Male C57BL/6J mice aged 8 weeks were purchased from Xi’an Jiaotong University (Xi’an, China) and housed in an environmentally controlled room under a 12 h/12 h light/dark cycle and at 22 ± 1 °C and 45 ± 5% relative humidity. There were eleven cages and three mice per cage. All animals were acclimated for 1 week on a standard maintenance diet (AIN 93M; TROPHIC Animal Feed High-Tech Co. Ltd. Nantong, China). Each mouse was randomly assigned to one of four diet groups for 14 weeks, and there were three cages per group. The first was a normal chow diet treatment (ND; 3.6 kcal/g; 10% energy from fat; AIN 93M). The second was a high-fat diet treatment (HFD; 4.5 kcal/g; 45% energy from fat; TP23100; TROPHIC Animal Feed High-Tech Co. Ltd., Nantong, China). The third was HFD with oral gavage of 400 mg/kg body weight (BW) GSE (HFD + GSE). The fourth was HFD with oral gavage of 400 mg/kg BW GPE (HFD + GPE). All mice had ad libitum food and water access during the experiment. It was previously established that the GSE and GPE dosages used in the present study fell within their respective no observed adverse effect level (NOAEL) ranges [30]. For the mice in the HFD + GSE and HFD+GPE groups, the GSE and GPE were dispersed in distilled water and administered by oral gavage every second day. The mice in the ND group and HFD group were administered equal volumes of distilled water by oral gavage every other day. The HFD components are listed in Supplementary Table S1.

Food intake and body weight were recorded weekly for the first 12 of the 14 weeks of the feeding trial. Metabolic measurements were made in Week 13. By the end of the experiment after 14 weeks, fighting and oral gavage injury had reduced the number of surviving mice to eight per group. The animals were fasted for 12 h and anesthetized. Blood was collected from their orbital plexuses and centrifuged at 3000 rpm and 4 °C for 15 min to isolate the serum. The serum samples from each mouse were separately sealed in test tubes and stored at −80 °C. The mice were euthanized by cervical dislocation, placed on ice, and aseptically dissected. The eWAT, iWAT, and interscapular BAT were excised and weighed. A small piece was removed from each adipose tissue and separately stored in 4% (*v*/*v*) paraformaldehyde (PFA) for histological analysis. The remaining pieces of each type of adipose tissue from each animal were then individually wrapped in sterile aluminum foil, frozen in liquid nitrogen, and stored at −80 °C. All experimental procedures were conducted according to the directives of the Guide for the Care and Use of Laboratory Animals (Eighth Edition; ISBN-10 No. 0-309-15396-4). The experimental protocols were approved by the Experimental Animal Ethics Committee of Northwest A&F University (Permission ID: 20200528-010, Date: 15 Nov 2020).

### 2.4. Energy Intake and Food Efficiency Ratio

The energy intake and food efficiency ratio (FER) were calculated based on the recorded food intake and body weight. Fresh high-fat feed was weighed (W_1_) and delivered to each cage at the same time each week. Any leftover feed (orts) was collected from each cage and weighed (W_2_) at the same time during the following week. The weekly food intake in each cage was calculated as follows:

ΔW = W_1_ − W_2_ (3)

ΔW was then divided by seven (days) and the number of mice in each cage to obtain the daily food intake in grams per mouse per day. The daily energy intake in kilocalories per mouse per day was the product of the daily food intake multiplied by the energy density of the high-fat feed, namely, 4.5 kcal/g.

The food efficiency ratio was the quotient of the weight gain (or difference between the weight at Week 12 and the baseline weight) divided by the total food intake over 12 weeks.

### 2.5. Metabolic Chamber Analyses

In the 13th week of the feeding experiment, one mouse was randomly selected from each cage (for a total of three mice per group) and placed in a metabolic chamber (Columbus Instruments, Columbus, OH, USA) to determine diurnal and nocturnal whole-body energy metabolism. Briefly, each mouse was individually housed in metabolic cages at 22 °C under a 12 h light/12 h dark cycle, had ad libitum food and water access, and was acclimated for 24 h. The oxygen consumption (VO_2_; mL/kg/h), carbon dioxide production (VCO_2_; mL/kg/h), respiratory exchange ratio (RER), and heat expenditure (kcal/h/g) were continuously monitored and recorded with sensors over the next 24 h period. The RER and heat expenditure were calculated as follows:

RER = VCO_2_/VO_2_ (4)

Heat expenditure = VO_2_ × [(1.232 × RER) + 3.815] (5)

### 2.6. Serum Profile Analysis

Eight serum samples were collected per group. The serum triglyceride, total cholesterol (TC), high-density lipoprotein cholesterol (HDL-C), and low-density lipoprotein cholesterol (LDL-C) levels were measured with assay kits (Nanjing Jiancheng Bioengineering Institute, Nanjing, China). Fasting serum glucose levels were measured with a glucose analyzer (Sinocare Inc., Changsha, China).

### 2.7. Histology Analysis

BAT, iWAT, and eWAT were fixed in 4% (*v*/*v*) PFA, embedded in paraffin, cut into sections 5 μm thick, and stained with hematoxylin and eosin (H&E) for morphological observations and adipocyte size analysis. Six mice were randomly selected from each group, and two sections of each type of adipose tissue were prepared per mouse. Two visual fields per slice were randomly selected and visualized and photographed under an optical microscope fitted with a camera (Olympus, Tokyo, Japan). ImageJ software (National Institutes of Health (NIH), Bethesda, MD, USA) was used to estimate the sizes of the adipocytes in the photographs.

### 2.8. RNA Isolation and Real-Time Quantitative Polymerase Chain Reaction (RT-qPCR)

Adipose tissues (BAT, iWAT, and eWAT) were randomly selected from six mice per group and the total RNA was extracted with a HiPure Universal RNA Kit (Magen Biotechnology Co. Ltd., Guangzhou, China). Total RNA purity and concentration were determined with a microvolume UV–Vis spectrophotometer (NanoDrop One; Thermo Fisher Scientific, Waltham, MA, USA). The total RNA was reverse-transcribed to cDNA with HiScript II Q RT SuperMix (R223-01; Vazyme, Nanjing, China). The latter was combined with ChamQ Universal SYBR qPCR Master Mix (Q711; Vazyme) and primers specific to mouse target genes. The qPCR was performed in a QuantStudio 6 Flex Real-Time PCR System (Thermo Fisher Scientific). The reaction conditions were as follows: initial denaturation stage, 95 °C for 30 s, one repetition; cycling reaction stage, 95 °C for 10 s followed by 60 °C for 30 s, 40 repetitions; and melting curve stage, 95 °C for 15 s, 60 °C for 60 s, and 95 °C for 15 s, one repetition. The cycle threshold (*C_t_*) values were standardized to the glyceraldehyde-3-phosphate dehydrogenase gene *(Gapdh*), and the relative mRNA expression levels of the target genes were evaluated by the 2^−ΔΔCt^ method. The target genes regulated lipogenesis (*Fas*, *Acc*, *Pparγ*, *Srebp1c*, *C/ebp-α*, *C/ebp-β*, *Ap2*), lipolysis (*Atgl*, *Hsl*, *Pparα*, *Cpt1-α*), thermogenesis (*Ucp1*, *Cidea*, *Dio2*, *Pgc1α*, *Pgc1β*, *Prdm16*, *Fgf21*, *Tgr5*), and mitochondrial biogenesis (*Tfam*, *Nrf1*, *Nrf2*). The primers used in the RT-qPCR are listed in Supplementary Table S2.

### 2.9. Statistical Analysis

All data generated by the GSE and GPE analyses are means ± standard deviation (SD), while those derived from the animal experiments are means ± standard error of the mean (SEM). Differences between means were determined by unpaired *t*-test or one-way analysis of variance (ANOVA). Multiple comparisons were made by Duncan’s test. *p* < 0.05 was considered statistically significant. Data were processed in SPSS v. 26.0 (SPSS Corp., Chicago, IL, USA), and graphs and figures were plotted with GraphPad Prism v. 9.0 (GraphPad Software Inc., San Diego, CA, USA).

## 3. Results

### 3.1. Phenolic Compounds in GSE and GPE

Dunkelfelder grape pomace was selected as the plant material for the present study as its seeds and peel had the highest total polyphenol content, and its peel had the highest total anthocyanin content of all eight red grape varieties analyzed in our laboratory (Supplementary Table S3). The lyophilized extracts of Dunkelfelder grape pomace seeds and peel were used in the animal experiments. The total phenolic, flavanol, flavonoid, tannin, and anthocyanin levels in GSE and GPE are listed in Table 1. In GSE, tannins are the most abundant, followed by flavonoids and flavanols. Tannins, also known as proanthocyanidins, are polymers with a degree of polymerization greater than 3, formed by condensation of flavan-3-alcohol monomers [31]. The total tannin content of GSE was much higher than that of GPE, which is consistent with the results reported by KY et al. [32]. The total flavonoid content of GPE was similar to that of tannin [32]. Anthocyanins are the color substances of the grape skin, not found in the seeds, and are therefore the characteristic components of GPE [31]. Overall, the total concentrations of all phenolic substances except anthocyanins were higher in GSE than GPE in this study.

The concentrations of the low-molecular-weight phenolic substances in GSE were measured by HPLC. Table 2 lists the top 10 most abundant phenolics. The flavan-3-ols had the highest concentration (138.71 μg/mg). It was previously reported that flavan-3-ols were the richest extractable phenolics in grape seed [31,33]. Epicatechin (49.31 μg/mg) and catechin (46.80 μg/mg) are the predominant flavan-3-ols monomer derivatives. Anthocyanins occur only in red grape skins. We therefore carried out HPLC analysis specifically on the GPE anthocyanin profiles. As shown in the list on the right of Table 2, malvidin-3-glucoside and malvidin-3-coumaroyl-glucoside were the two most abundant anthocyanins in GPE (30.02 μg/mg and 28.50 μg/mg, respectively).

### 3.2. GSE and GPE Reduced Body Weight and Normalized Blood Constituents in HFD-Fed Mice

**Figure 1:**
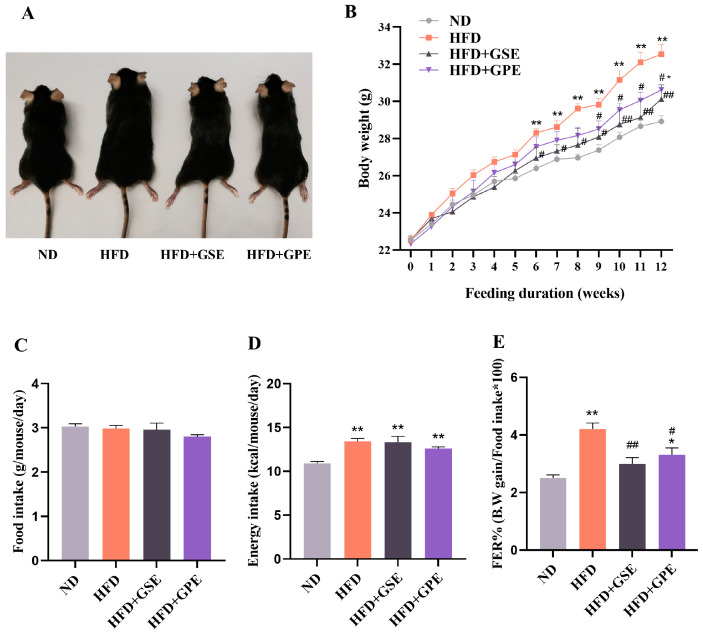
Effects of GSE and GPE on body weight gain and energy intake in HFD-fed mice. (**A**) Representative mice of each group. (**B**) Body weight. (**C**) Food intake. (**D**) Energy intake. (**E**) Food efficiency ratio (FER). Results are presented as mean and SEM of three independent experiments, * *p* < 0.05, ** *p* < 0.01 versus the ND group, # *p* < 0.05, ## *p* < 0.01 versus the HFD group.

Male 9-week C57BL/6J mice similar in initial body weight were randomly divided into four groups: the ND group on a normal chow diet and the HFD group on a high-fat diet. For the experimental treatments, the mice were fed HFD and given 400 mg/kg body weight GSE or GPE by oral gavage. The effects of GSE and GPE on body weight in mice are shown in Figure 1A,B. From Week 6 onwards, the HFD group showed significantly higher body weight than the ND group (*p* < 0.01). At the same time, the HFD + GSE group presented with significantly lower body weight (*p* < 0.05) than the HDF group. Marked weight loss began at Week 9 in the HFD+GPE group (*p* < 0.05) compared to the HFD group. By Week 12 of the feeding trial, body weight gain was 24.5% and 17.3% lower in the GSE- and GPE-treated mice than in the HFD group, respectively, and the body weight of the GSE group was closer to that of the ND group (*p* > 0.05) than that of the GPE group (*p* < 0.05).

We then explored whether the observed effects of GSE and GPE on body weight were associated with food intake. The food and energy intake levels were comparable for the HFD + GSE, HFD + GPE, and HFD groups (*p* > 0.05) (Figure 1C,D). Nevertheless, the food efficiency ratios (FERs) were significantly lower for the HFD + GSE and HFD + GPE groups than the HFD group (Figure 1E). Hence, GSE and GPE inhibited high-fat diet-induced body weight gain in an appetite-independent manner.

Prolonged consumption of a high-fat diet negatively affects serum biochemistry. As shown in Table 3, HFD resulted in significantly higher serum levels of TC, TG, LDL, and HDL than those in the ND group. However, both GSE and GPE had similar efficacy in improving the serum lipid parameters of the mice on a high-fat diet. GSE and GPE markedly decreased TG and TC and increased HDL-C but did not affect LDL. The mice administered GSE and GPE had significantly lower fasting serum glucose levels than those in the HFD group.

The preceding results demonstrated that GSE and GPE supplementation reversed HFD-induced weight gain and abnormal blood parameters. The obesity and metabolic impairments caused by HFD are closely associated with adipose tissue dysfunction. Thus, we investigated the effects of GSE and GPE supplementation on adipose tissue.

### 3.3. GSE and GPE Reduced Adipose Tissue Weight and Adipocyte Size in HFD-Fed Mice

**Figure 2:**
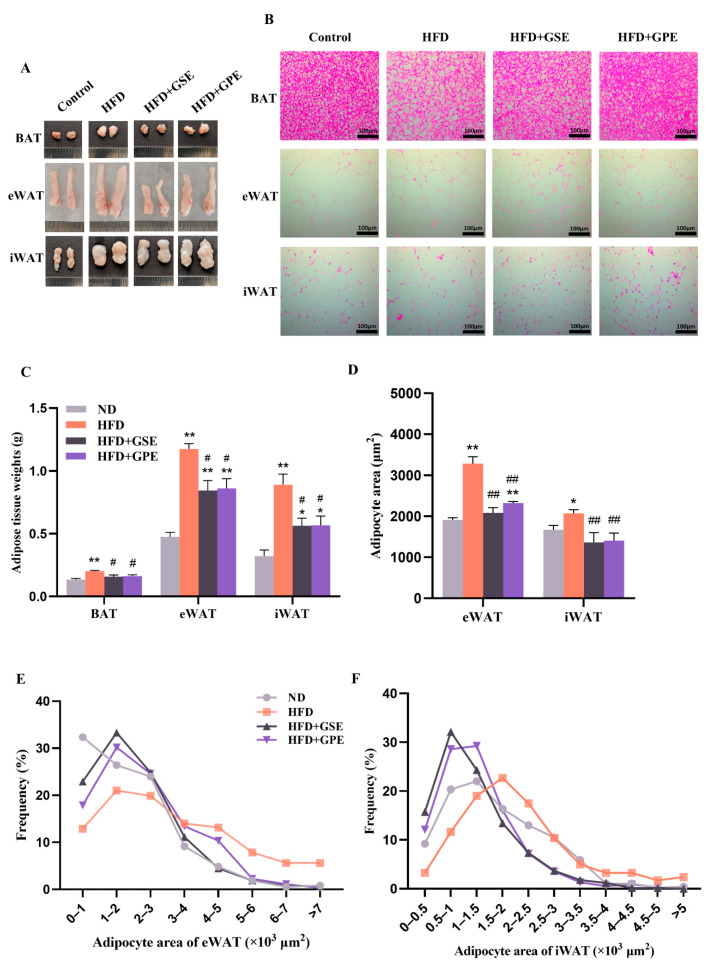
Effects of GSE and GPE on adipose tissue weight gain and adipocyte size in HFD-fed mice. (**A**) Representative adipose tissue of each group. (**B**) Representative images of H&E staining of adipose tissue. (**C**) Adipose tissue weight. (**D**) Average sizes of adipocytes in eWAT and iWAT. (**E**) Adipocyte size distribution in eWAT. (**F**) Adipocyte size distribution in iWAT. Results are presented as mean and SEM of three independent experiments, * *p* < 0.05, ** *p* < 0.01 versus the ND group, # *p* < 0.05, ## *p* < 0.01 versus HFD group.

The effects of GSE and GPE on HFD-induced adipocyte hypertrophy were examined by determining the changes in BAT, eWAT, and iWAT weight. The adipose tissues were stained with H&E, and adipocyte size and distribution frequency were determined. Representative adipose tissues for each group are shown in Figure 2A. By Week 14 of the feeding trial, the BAT, eWAT, and iWAT weights in mice in the HFD group increased significantly compared with those in the ND group. However, the weights of these three adipose tissues were markedly lower in the mice supplemented with GSE or GPE than in those on the HFD, although their eWAT and iWAT weights did not recover to the levels of those in the ND group (Figure 2C). Histological analyses showed that the sizes of the lipid droplets in the BAT and the average areas of the adipocytes in the eWAT and iWAT were smaller in the GSE- and GPE-treated mice than they were in the HFD mice (Figure 2B,D). In the eWAT of mice in the HFD, HFD + GSE, and HFD + GPE groups, adipocytes with an area of between 1000 and 2000 μm^2^ were most abundant. The proportions of adipocytes within this range were significantly higher for the GSE- and GPE-treated mice than the HFD mice. The latter presented with large numbers of adipocytes >2000 μm^2^ in area (Figure 2E). In the iWAT, the maximum number of adipocytes was distributed in the range of 500–1000 μm^2^ cell area in both HFD + GSE and HFD + GPE mice. In contrast, the adipocytes with an area of between 1000 and 2000 μm^2^ were most abundant in the iWAT for the HFD mice (Figure 2F). The foregoing results suggest that the GSE and GPE treatments attenuate HFD-induced lipid droplet accumulation in adipose tissues and can, therefore, improve lipid metabolism and promote body weight reduction.

### 3.4. Effects of GSE and GPE on the Expression Levels of the Genes Regulating Lipid Metabolism in the Adipose Tissues of HFD-Fed Mice

**Figure 3:**
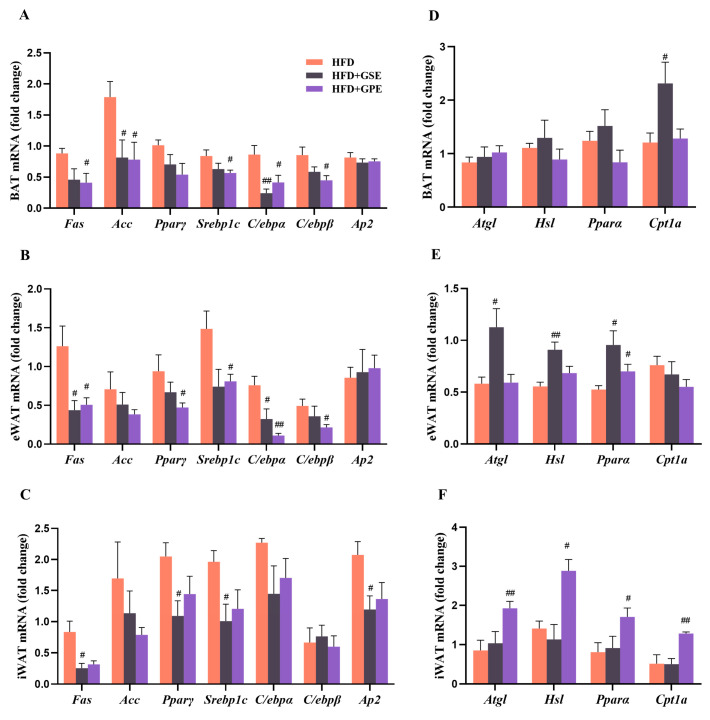
Effects of GSE and GPE on the expression levels of the genes regulating lipid metabolism in the adipose tissues of HFD-fed mice. (**A**–**C**) Lipogenic gene mRNA levels in BAT, eWAT, and iWAT. (**D**–**F**) Lipolytic gene mRNA levels in BAT, eWAT, and iWAT. Results are presented as mean and SEM of three independent experiments, # *p* < 0.05, ## *p* < 0.01 versus HFD group.

The GSE and GPE treatments inhibited HFD-induced lipid accumulation. Thus, we endeavored to determine whether these treatments modulated the expression of genes controlling lipid metabolism in all three adipose tissues. Compared to the HFD group, the BAT of the HFD + GSE group presented with downregulated *Acc* and *C/ebpα*. The HFD+GPE group exhibited reduced expression of *Fas*, *Acc*, *Srebp1c*, *C/ebpα*, and *C/ebpβ* (Figure 3A). Only the GSE treatment increased the *Cpt1α* mRNA levels in the BAT of mice on a high-fat diet (Figure 3D).

The eWAT of the HFD + GSE group had lower *Fas* and *C/ebpα* and higher *Atgl*, *Hsl*, and *Pparα* expression levels than that of the HFD group. GPE supplementation significantly decreased the relative lipogenesis gene (*Fas*, *Pparγ*, *Srebp1c*, *C/ebpα*, and *C/ebpβ*) mRNA levels in the eWAT of HFD-fed mice. In contrast, the GPE treatment only increased the relative mRNA level of the lipolytic gene *Pparα* (Figure 3B,E).

In the iWAT of the HFD+GSE group, *Fas*, *Pparγ*, *Srebp1c*, and *Ap2* were markedly downregulated, whereas the relative expression levels of the lipolytic genes did not substantially change (Figure 3C,F). In the HFD + GPE group, the mRNA levels of *Atgl*, *Hsl*, *Pparα*, and *Cpt1α* were significantly increased, and the relative mRNA levels of lipogenic genes showed a decreasing trend (not statistically significant).

The preceding results suggested that GSE and GPE supplementation partially reverse lipid metabolism dysregulation of adipose tissues induced by a high-fat diet. GSE supplementation significantly downregulated the genes regulating lipid biosynthesis in the BAT, eWAT, and iWAT of HFD-fed mice, and upregulated the lipolytic genes in the eWAT. In contrast, GPE supplementation substantially decreased the relative expression levels of the lipid biosynthesis genes in the BAT and eWAT, and upregulated the lipolytic genes in the iWAT.

### 3.5. GSE and GPE Improved Energy Expenditure in HFD-FED mice

Nutrient and energy metabolism occur simultaneously in living organisms. The preceding results showed that the GSE and GPE treatments improved lipid metabolism in the adipose tissue of mice on a high-fat diet. Therefore, we investigated whether the GSE and GPE treatments could ameliorate energy expenditure in HFD-fed mice in metabolic chambers.

**Figure 4:**
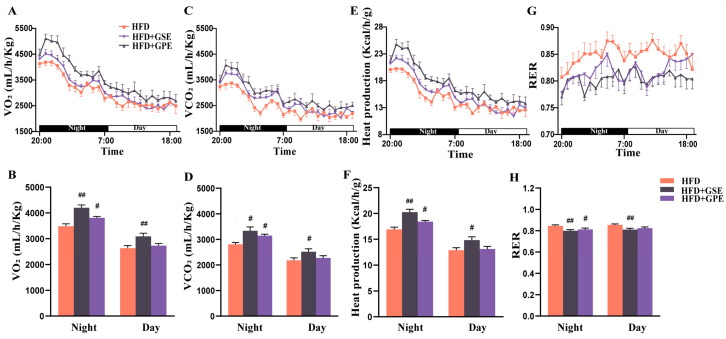
Effects of GSE and GPE supplementation on energy expenditure in HFD-fed mice. Oxygen consumption (VO_2_), carbon dioxide release (VCO_2_), heat production, and respiratory exchange ratio (RER) were detected during a 12 h light/dark cycle in HFD-fed mice in metabolic chambers (**A**,**C**,**E**,**G**). Bar graphs represent the averages for nocturnal (active stage) and diurnal (quiescent stage) VO_2_, VCO_2_, heat production and RER (**B**,**D**,**F**,**H**). Results are presented as mean and SEM of three replicates, # *p* < 0.05, ## *p* < 0.01 versus the HFD group.

Figure 4 4A–F show that for all treatment groups, O_2_ consumption (VO_2_), CO_2_ release (VCO_2_), and heat production were higher at nighttime than in the daytime as mice are nocturnal mammals. After GSE or GPE supplementation, nocturnal VO_2_, VCO_2_, and heat production markedly increased in the mice on a high-fat diet. Whereas the GSE treatment also augmented the foregoing parameters in the daytime, the GPE treatment had no such effect.

The type of fuel consumed for energy production may be inferred from the respiratory exchange rate (RER). Sugar-based and lipid-based energy metabolism pathways are associated with RER ~1 and RER ~0.7, respectively [34]. Here, GSE supplementation significantly reduced both the nocturnal and diurnal RER of the mice on a high-fat diet. In contrast, GPE supplementation only lowered the nocturnal RER of the HFD-fed mice (Figure 4G–H). The preceding results suggest that both the GSE and GPE treatments increased the proportions of lipids being consumed for energy production and promoted body fat utilization in the mice on a high-fat diet.

Comprehensive analyses of the foregoing results and the energy intake levels (Figure 1C) demonstrated that both the GSE and GPE treatments resisted HFD-induced obesity by enhancing energy expenditure and increasing the proportion of lipids being consumed for energy production without affecting energy intake. Furthermore, GSE supplementation more effectively increased the basal metabolic rates of the HFD-fed mice than GPE supplementation based on the relative diurnal (quiescent stage) VO_2_, VCO_2_, and RER of these groups.

### 3.6. Effects of GSE and GPE on Expression Levels of Genes Regulating Thermogenesis and Mitochondrial Biogenesis in Adipose Tissues of HFD-Fed Mice

**Figure 5:**
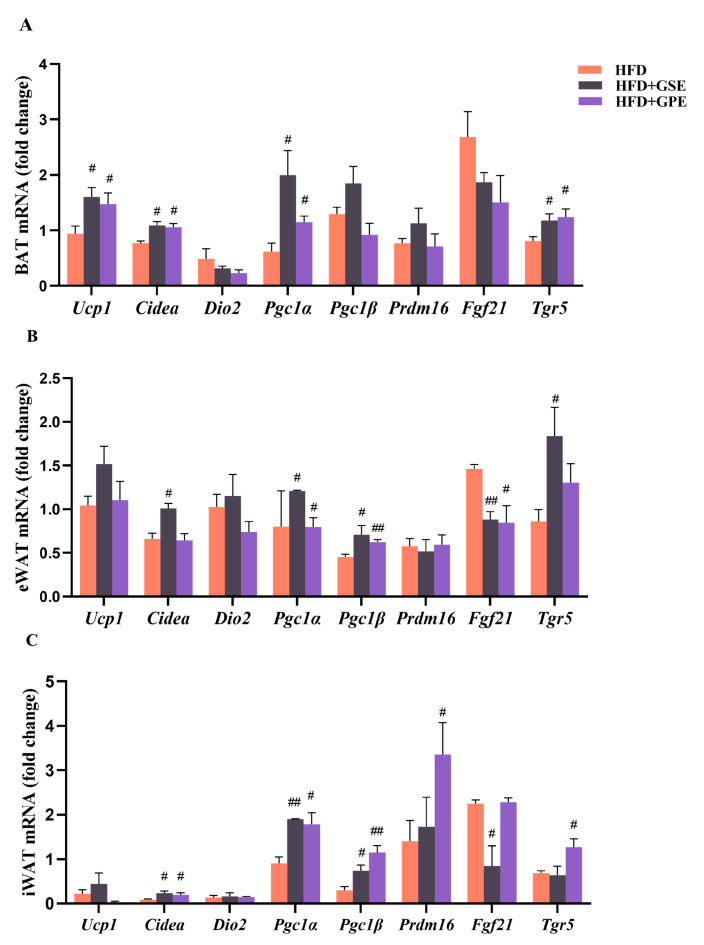
Effects of GSE and GPE on the expression levels of the thermogenic genes in the adipose tissue of HFD-fed mice. (**A**) Thermogenic gene mRNA expression levels in BAT. (**B**–**C**) Browning-related gene mRNA expression levels in eWAT (**B**) and iWAT (**C**). Results are presented as mean and SEM of three independent experiments, # *p* < 0.05, ## *p* < 0.01 versus HFD group.

Adipose tissue-mediated non-shivering thermogenesis is a vital energy dissipation mechanism that animals utilize to maintain their core body temperature [35]. BAT is the principal thermogenic adipose tissue. The beige adipocytes produced by adipocyte ‘browning’ in white adipose tissue are also thermogenic [36]. Activation of these thermogenic mechanisms helps ameliorate diet-induced obesity [9]. We measured the expression levels of the thermogenesis-related genes in BAT and browning WAT. We aimed to establish whether the mechanisms by which GSE and GPE improve obesity and energy metabolism-related disorders are associated with adipose tissue thermogenesis in HFD-fed mice. Figure 5A shows that compared to the HFD treatment, the GSE and GPE treatments upregulated *Ucp1*, *Cidea*, *Pgc1α*, and *Tgr5* in the BAT. In the eWAT (Figure 5B) and iWAT (Figure 5C), the GSE treatment upregulated *Cidea*, *Pgc1α*, and *Pgc1β* and downregulated *Fgf21* relative to the HFD treatment. Furthermore, GSE supplementation upregulated *Tgr5* in the eWAT, while GPE supplementation upregulated *Pgc1α* and *Pgc1β* and downregulated *Fgf21* in the eWAT (Figure 5B). GPE supplementation also upregulated *Cidea*, *Pgc1α*, *Pgc1β*, *Prdm16*, and *Tgr5* in the iWAT (Figure 5C).

**Figure 6:**
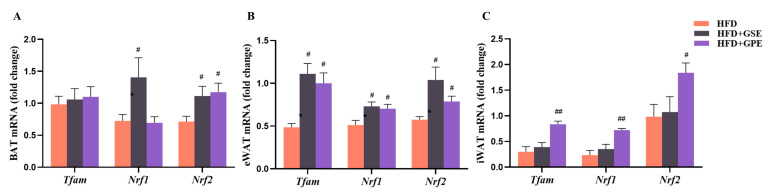
Effects of GSE and GPE on mitochondrial biogenesis-associated gene expression in the adipose tissue of HFD-fed mice. (**A**–**C**) Expression profiles of mitochondrial biogenesis-related genes in BAT (**A**), eWAT (**B**), and iWAT (**C**). Results are presented as mean and SEM of three independent experiments, # *p* < 0.05, ## *p* < 0.01 versus HFD group.

The activation of BAT and beige adipocyte formation in WAT may be positively correlated with enhanced mitochondrial biogenesis and function [37,38]. Thus, we measured the mRNA expression levels of mitochondriogenesis-related genes in all three adipose tissues. The GSE treatment upregulated *Nrf1* and *Nrf2* in the BAT compared to the HFD treatment (Figure 6A). The GPE treatment also partially promoted mitochondrial biogenesis by upregulating *Nrf2* in the BAT. The GSE treatment upregulated *Tfam*, *Nrf1*, and *Nrf2* expression in the eWAT (Figure 6B), but it had no significant effect on the expression levels of the genes regulating mitochondriogenesis in the iWAT of mice on a high-fat diet (Figure 6C). In contrast, the GPE treatment greatly enhanced *Tfam* and *Nrf1/2* expression in both the eWAT and the iWAT of the HFD-fed mice.

Taken together, the preceding data showed that GSE or GPE supplementation has the potential to promote BAT thermogenesis, eWAT and iWAT browning, and by extension, enhanced energy dissipation in mice on a high-fat diet. Specifically, the GSE or GPE supplementation could augment the expression of the genes regulating BAT thermogenesis and WAT browning. Furthermore, GSE supplementation promoted mitochondrial biogenesis only in BAT and eWAT, whereas GPE supplementation enhanced it in all three adipose tissues.

## 4. Discussion

In the present study, we elucidated the energy metabolism-related mechanism by which grape seed extract ameliorates adipose tissue dysregulation caused by a high-fat diet. We also discovered that grape peel extract could prevent obesity by a mechanism somewhat similar to that of grape seed extract. The specific findings are as follows: (1) GSE or GPE supplementation reversed weight gain and blood parameter disturbances in mice maintained on a high-fat diet. (2) GSE or GPE supplementation promoted body fat utilization by modulating the expression of the genes regulating lipid metabolism in adipose tissue. In this manner, GSE or GPE decreased adipose tissue mass and lipid droplet accumulation in adipocytes. (3) GSE or GPE enhanced energy expenditure by upregulating the genes associated with BAT thermogenesis and WAT browning as well as those controlling mitochondrial biogenesis.

Both GSE and GPE reduced HFD-induced weight gain in mice. This finding is consistent with those reported in previous studies [39,40]. Nevertheless, inhibition of the HFD-induced weight effect occurred earlier in response to the GSE treatment than the GPE treatment (Figure 1B). One possible explanation for this discrepancy is the fact that grape seeds contain higher phenolic concentrations than grape peel (Table 1). Both the GSE and GPE treatments lowered the fasting serum glucose, TG, and TC levels and raised the serum HDL-C levels in mice fed a high-fat diet (Table 3).

Excessive fat accumulation in the body in general, and the adipose tissue in particular, is the most prominent feature of obesity [41]. This condition is characterized by increases in the number and size of adipocytes resulting from imbalances in lipid and energy metabolism in adipose tissues. The results of the present study demonstrated that GSE or GPE treatment could partially reverse the aforementioned dysfunctions. Oxygen consumption and carbon dioxide production were significantly higher in the HFD + GSE and HFD + GPE groups than in the HFD group (Figure 4A–D). It indicates that GSE or GPE supplementation augmented oxidation of fuel substances in mice. Moreover, the data for the daytime (resting period) confirmed that GSE markedly improved the basal metabolic rate in the HFD mice. This was consistent with the relatively greater weight loss in the HFD + GSE group. The RER is a vital clinical indicator of the type of fuel consumed for energy generation and is derived from the VCO_2_: VO_2_ ratio. Here, the RER was lower for the HFD + GSE and HFD + GPE groups than the HDF group (Figure 4G–H). Thus, the GSE and GPE treatments increased the proportion of lipids consumed in metabolic energy production in the mice on a high-fat diet. The preceding results imply that GSE or GPE supplementation promoted body fat utilization and beneficially reduced lipid accumulation in the adipocytes. This conclusion was validated by the morphological analyses of the adipose tissue sections (Figure 2C–F) as well as upregulation of the lipid metabolism-related genes in them (Figure 3). In mice on a high-fat diet, supplementation with GSE significantly reduced the expression of lipogenic genes in BAT (*Acc*, *C/ebpα*), eWAT (*Fas*, *C/ebpα*) and iWAT (*Fas*, *Pparγ*, *Srebp1c*, *Ap2*), and increased the expression of lipolytic genes in BAT (*Cpt1α*) and eWAT (*Atgl*, *Hsl*, *Pparα*). The HFD+GPE group had a similar effect, but the adipose tissues and genes acted on were not identical to those of the HFD+GSE group, as evidenced by the downregulation of expression of lipogenic genes in BAT (*Fas*, *Acc*, *Srebp1c*, *C/ebpα*, *C/ebpβ*) and eWAT (*Fas*, *Pparγ*, *Srebp1c*, *C/ebpα*, *C/ebpβ*) and upregulation of expression of genes controlling fatty acid oxidation in eWAT (*Pparα*) and iWAT (*Atgl*, *Hsl*, *Pparα*, *Cpt1α*).

Heat production was significantly enhanced in the HFD-fed mice administered GSE or GPE (Figure 4E–F) possibly because energy generation increased in response to augmented fuel oxidation. High metabolic energy expenditure may be associated with thermogenesis in BAT and browning in WAT. Both the GSE and GPE treatments upregulated *Ucp1*, *Cidea*, and *Pgc1α* in the BAT of HFD-fed mice (Figure 5A). In the inner mitochondrial membrane, UCP1 uncouples mitochondrial respiration from ATP biosynthesis, thereby dissipating thermal energy [8]. The coactivator peroxisome proliferator-activated receptor coactivator 1 α (PGC1α) can induce UCP1 [42]. Cell death-inducing DFFA-like effector A (CIDEA) is a marker of the thermogenic capacity of brown and beige adipocytes. In mice supplemented with GSE, browning was promoted in WAT (Figure 5B–C), and the mRNA expression levels of *Cidea*, *Pgc1α*, and *Pgc1β* in their eWAT and iWAT were higher than those of the mice on a high-fat diet. In mice supplemented with GPE, the mRNA levels of *Pgc1α* and *Pgc1β* in their eWAT and iWAT and *Cidea* and *Prdm16* in their iWAT were higher than those in the same tissues of the mice on a high-fat diet. *PGC1α* and *Pgc1β* play important roles in mitochondrial biogenesis [43]. The transcription factor (TF) PRDM16 promotes the differentiation of brown and beige adipocytes [44].

The GSE and GPE-induced healthier metabolisms mentioned above are closely linked to their phenolic composition. Previous reports have shown that grape seed proanthocyanidins activated adipose thermogenesis [45], and ameliorated inflammation and adiposity in high-fat diet mice [46]. It also reduced adipocyte size and increased adipocyte number [18]. Moreover, daily ingestion of catechin-rich beverages enhanced brown adipose tissue density and decreased extramyocellular lipids in healthy young women. Additionally, anthocyanins significantly reduced serum TC, TG, and LDL-C levels and elevated HDL-C in patients with dyslipidemia [47], and long-term supplementation could reduce body mass index (BMI) and body weight [48].

Fibroblast growth factor 21 (FGF21) is an endocrine hormone produced mainly by the liver and adipocytes [49]. It activates brown adipose tissues, induces thermogenic gene expression, and causes the appearance of brown-like adipocytes in WAT [50]. Though FGF21 is promising as an anti-obesity agent, its relationship with obesity remains controversial. Al-Amrani et al. reported that certain obese patients had high circulating FGF21 levels and impaired FGF21 signaling. Hence, obesity might be associated with FGF21 resistance [51]. Figure 5 shows that GSE supplementation significantly downregulated *Fgf21* in the eWAT (*p* < 0.01) and iWAT (*p* < 0.05), whereas GPE supplementation downregulated *Fgf21* in the eWAT (*p* < 0.05) of HFD-fed mice. Thus, GSE or GPE supplementation could have the potential to ameliorate FGF21 resistance in obese patients.

We also discovered that in HFD-fed mice, GSE supplementation increased the mRNA expression levels of *Tgr5* in BAT (*p* < 0.05) and eWAT (*p* < 0.05), while GPE supplementation increased the mRNA levels of *Tgr5* in BAT (*p* < 0.05) and iWAT (*p* < 0.05) (Figure 5). G protein-coupled receptor 5 (TGR5) binds bile acids and regulates UCP1 by activating type 2 deiodinase (DIO2) in BAT [52,53]. It also causes browning in subcutaneous WAT by inducing mitochondrial fission, β-oxidation, and thermogenic activity [54]. Therefore, we suspect that the activation of the thermogenic capacity of adipose tissue by GSE and GPE treatment in mice on a high-fat diet is likely to be related to bile acid metabolism. This hypothesis was partially validated by Han et al. [39], and still, it needs to be further investigated.

In adipose tissue, lipid oxidation and heat production are positively correlated with enhanced mitochondrial biogenesis and function. Mitochondrial transcription factor A (TFAM) is a major regulator of mitochondrial DNA replication and transcription [55]. Nuclear respiratory factor 1 (NRF1) forms a coactivator with PGC-1 to activate the TFAM promoter and stimulate TFAM activity [56]. NRF1 and NRF2 are also involved in regulating the transcription of genes associated with the mitochondrial electron transport chain [57]. These transcription factors modulate cellular respiration and energy metabolism by affecting mitochondrial function and quantity. Here, the GSE and GPE treatments upregulated the genes controlling mitochondrial biogenesis in adipose tissue (Figure 6). GSE supplementation increased the mRNA expression levels of *Nrf1* and *Nrf2* in both BAT and eWAT and *Tfam* in eWAT. In contrast, GPE supplementation increased the mRNA expression levels of *Tfam*, *Nrf1*, and *Nrf2* in both eWAT and iWAT and *Nrf2* in BAT.

There are several limitations in this study. Although the present study has demonstrated an ameliorative effect of GSE and GPE on obesity, it is unclear whether weight loss is primarily contributed by a single bioactive ingredient or caused by synergistic effects with multiple ingredients. Therefore, future research should concentrate on screening the bioactive components of GSE and GPE to investigate their anti-obesity benefits singly or in conjunction with other components. In addition, only the mRNA expression levels of key genes were examined in this study. If the detection of a protein level can be supplemented, the conclusion will be more convincing.

## 5. Conclusions

The present work disclosed that while the GSE and GPE treatment did not affect energy intake in mice on a high-fat diet, they could achieve an upregulation of the proportion of lipids in energy-supplying substrates, which suggested more fatty acid oxidation in mice. This was consistent with the fact that both GSE and GPE increased the mRNA expression levels of the genes regulating lipolysis and decreased those of the genes regulating lipogenesis in adipose tissue. They also improved calorie dissipation in HFD-fed mice by upregulating the genes controlling thermogenesis in BAT, browning in eWAT and iWAT, and mitochondrial biogenesis in all three types of adipose tissue. The foregoing results demonstrated that GSE and GPE augment body fat utilization by increasing lipid and energy metabolism. In this manner, they can attenuate high-fat diet-induced lipid accumulation and weight gain and normalize blood glucolipid levels. The promising findings of this study suggest that GSE and GPE could be administered as nutraceuticals complementing other prophylactic and therapeutic strategies applied against obesity-related co-morbidities.

## Figures and Tables

**Table 1:** Total phenolic, flavonoid, flavanol, tannin, and anthocyanin content in GSE and GPE.

	TPC (GAE mg/g)	TANC (CE mg/g)	TFOC (RE mg/g)	TFAC (CE mg/g)	TAC (C3GE mg/g)
GSE	656.40 ± 8.07	680.62 ± 3.82	271.20 ± 0.85	147.64 ± 1.78	-
GPE	173.70 ± 6.22	109.89 ± 1.66	105.53 ± 0.55	14.76 ± 0.54	75.46 ± 1.88

TPC, total polyphenol content; TANC, total tannin content; TFOC, total flavonoid content; TFAC, total flavanol content; TAC, total anthocyanin content. The results are expressed as milligrams equivalents of the respective standard per gram of GSE or GPE. Values are mean ± SD values of three replicates. GAE, gallic acid equivalents; CE, (+)-catechin equivalent; RE, rutin equivalent; C3GE, cyanidin 3-glucoside equivalent.

**Table 2:** Phenolic profiles of GSE and anthocyanin profiles of GPE.

Phenolic Profiles of GSE (μg/mg)		Anthocyanin Profiles of GPE (μg/mg)	
Flavan-3-ols		Malvidin-3-glucoside	30.02 ± 0.01
Epicatechin	49.31 ± 0.03	Malvidin-3-coumaroyl-glucoside	28.50 ± 0.02
Catechin	46.80 ± 0.02	Malvidin-3-acetyl-glucoside	6.43 ± 0.00
Gallocatechin	0.25 ± 0.01	Cyanidin-3-glucoside	2.90 ± 0.01
Epigallocatechin	0.12 ± 0.00	Peonidin-3-acetyl-glucoside	1.94 ± 0.01
Procyanidin B1	19.41 ± 0.02	Peonidin-3-coumaroyl-glucoside	1.86 ± 0.00
Procyanidin C1	16.81 ± 0.01	Delphinidin-3-glucoside	1.60 ± 0.01
Procyanidin B2	6.02 ± 0.01	Peonidin-3-glucoside	1.45 ± 0.00
Phenolic acids and Flavonols		Petunidin-3-glucoside	0.64 ± 0.00
Gallic acid	8.47 ± 0.02	Total	77.66 ± 0.09
Epicatechin-3-gallate	4.98 ± 0.01		
Quercetin-glucoside	0.12 ± 0.01		
Total	152.64 ± 0.07		
Sum of flavan-3-ols	138.71 ± 0.05 (91%)		

The phenolic profiles of GSE and anthocyanin profiles of GPE were determined by HPLC. Values are mean ± SD values of two replicates.

**Table 3:** GSE- and GPE-mediated improvements in the blood constituent levels of mice on a high-fat diet.

	ND	HFD	HFD + GSE	HFD + GPE
Triglyceride (mmol/L)	0.71 ± 0.13 b	1.03 ± 0.20 a	0.53 ± 0.09 b	0.66 ± 0.15 b
Total cholesterol (mmol/L)	2.43 ± 0.21 c	4.10 ± 0.65 a	3.49 ± 0.30 b	3.21 ± 0.19 b
HDL cholesterol (mmol/L)	3.24 ± 0.40 c	3.95 ± 0.15 b	4.57 ± 0.32 a	4.57 ± 0.33 a
LDL cholesterol (mmol/L)	0.16 ± 0.04 b	0.50 ± 0.16 a	0.43 ± 0.16 a	0.41 ± 0.12 a
Fasting serum glucose (mmol/L)	5.28 ± 0.61 b	6.92 ± 0.65 a	5.85 ± 0.64 b	6.04 ± 0.44 b

Results are presented as mean and SD values of three independent experiments. Mean values with different letters are significantly different (*p* < 0.05).

## Data Availability

The data presented in this study are available in the article.

## Supplementary Materials

The following supporting information can be downloaded at: https://www.mdpi.com/article/10.3390/foods12173251/s1, Table S1: Ingredients of high-fat diet; Table S2: Primer sequences used for semi-quantitative real-time PCR analysis; Table S3: Total phenolic, flavonoid, flavanol, tannin, and anthocyanin content of grape pomace from 8 varieties of red grapes (*Vitis vinifera*) after making dry red wine.
