# Supplementary material for: Extracts of Dunkelfelder Grape Seeds and Peel Increase the Metabolic Rate and Reduce Fat Deposition in Mice Maintained on a High-Fat Diet
Source: Foods. 2023 Aug 29;12(17):3251. doi: 10.3390/foods12173251 (PMC10487004; doi:10.3390/foods12173251)
Supplement: Supplementary file 1 [file foods-12-03251-s001.zip › foods-2553715-supplementary.pdf]

Supplementary Table 1 Ingredients of high fat diet

Ingredients	g/kg
Casein	175
Corn Starch	132
Maltodextrin	125
Sucrose	202
Soybean Oil	30
Lard	196
Cellulose	62
Mineral Mix, M1021	61
Vitamin Mix, V1010	12
L-Cystine	2
Choline Bitartrate	3
TBHQ	0.045

Supplementary Table 2 Primer sequences used for semiquantitative Real-time PCR analysis

	Forward primer	Reverse primer
Fas	AGGTGGTGATAGCCGGTATGT	TGGGTAATCCATAGAGCCCAG
Acc	CCGATTCATAATTGGGTCTGTGT	CCATCCTGTAAGCCAGAGATCC
Pparγ	TCGCTGATGCACTGCCTATG	GAGAGGTCCACAGAGCTGATT
Srebp1c	AGCCTGGCCATCTGTGAGAA	CAGACTGGTACGGGCCACAA
C/ebpα	TGGACAAGAACAGCAACGAG	TCACTGGTCAACTCCAGCAC
C/ebpβ	AAGCTGAGCGACGAGTACAAGA	GTCAGCTCCAGCACCTTGTG
Ap2	AAGAAGTGGGAGTGGGCTTTG	CTCTTCACCTTCCTGTCGTCTG
Atgl	TTCGCAATCTCTACCGCCTC	AAAGGGTTGGGTTGGTTCAG
Hsl	AGACCACATCGCCCACA	CCTTTATTGTCAGCTTCTTCAAGG
Pparα	AGCCTCAGCCAAGTTGAAGT	TGGGGAGAGAGGACAGATGG
Cpt1α	AGATCAATCGGACCCTAGACAC	CAGCGAGTAGCGCATAGTCA
Ucp1	AGGCTTCCAGTACCATTAGGT	CTGAGTGAGGCAAAGCTGATTT
Cidea	TGCTCTTCTGTATCGCCCAGT	GCCGTGTTAAGGAATCTGCTG
Dio2	CTTCCTCCTAGATGCCTACAAAC	GGCATAATTGTTACCTGATTCAGG
Pgc1α	TATGGAGTGACATAGAGTGTGCT	CCACTTCAATCCACCCAGAAAG
Pgc1β	CGTATTTGAGGACAGCAGCA	TACTGGGTGGGCTCTGGTAG
Prdm16	CCTCGCCATGTGTCAGATCAA	TTTCACATGCACCAACAGTTCC
Fgf21	ATGGAATGGATGAGATCTAGAGTTGG	TCTTGGTCGTCATCTGTGTAGAGG
Tgr5	CAGTCTTGGCCTATGAGCGT	CTGCCCAATGAGATGAGCGA
Tfam	ATTCCGAAGTGTTTTTCCAGCA	TCTGAAAGTTTTGCATCTGGGT
Nrf1	CGGAAACGGCCTCATGTGT	CGCGTCGTGTACTCATCCAA
Nrf2	TAGATGACCATGAGTCGCTTGC	GCCAAACTTGCTCCATGTCC
Gapdh	AGGTCGGTGTGAACGGATTTG	GGGGTCGTTGATGGCAACA

Supplementary Table 3 Total phenolics, flavonoids, flavanols, tannin and anthocyanins content of grape pomace from 8 varieties of red grapes (V*itis vinifera*) after making dry red wine.

	Seeds				Peels				
Varieties	TPC (GAE mg/g)	TFAC (CE mg/g)	TFOC (RE mg/g)	TANC (CE mg/g)	TPC (GAE mg/g)	TFAC (CE mg/g)	TAC (C3GE mg/g)	TFOC (RE mg/g)	TANC (CE mg/g)
Pinot Noir	123.09	69.17	258.35	81.53	23.01	6.52	0.70	37.80	6.78
Meili	83.49	62.52	198.82	61.86	11.23	3.45	0.52	16.36	5.35
Marselan	102.61	63.15	222.92	102.37	20.42	4.80	5.25	32.38	8.19
Merlot	88.74	54.70	207.53	77.72	32.04	9.29	3.13	48.84	5.46
Garanior	109.83	54.38	233.58	86.74	17.34	4.57	2.54	28.13	8.27
Cabernet Franc	77.47	42.09	165.78	68.73	17.71	6.51	0.85	29.47	11.59
Cabernet Sauvignon	77.17	36.87	142.71	59.64	25.65	8.56	2.41	39.48	10.44
**Dornfelder**	**130.50**	**68.13**	**257.80**	**97.53**	**54.93**	**10.55**	**20.54**	**69.70**	**31.69**

GAE mg/g, RE mg/g, CE mg/g, and C3GE mg/g represent milligrams of gallic acid equivalents, milligrams of rutin equivalent, milligrams of (+)-catechin equivalent, and milligrams of cyanidin 3-glucoside equivalent per gram of dry grape seed or peel, respectively.
